# Assessment of Autonomic Nervous System Function in Patients with Chronic Fatigue Syndrome and Post-COVID-19 Syndrome Presenting with Recurrent Syncope

**DOI:** 10.3390/jcm14030811

**Published:** 2025-01-26

**Authors:** Branislav Milovanovic, Nikola Markovic, Masa Petrovic, Vasko Zugic, Milijana Ostojic, Ljiljana Rankovic-Nicic, Milovan Bojic

**Affiliations:** 1 Institute for Cardiovascular Diseases “Dedinje”, 11000 Belgrade, Serbia; prof.dr.branislavmilovanovic@gmail.com (B.M.);; 2 Faculty of Medicine, University of Belgrade, 11000 Belgrade, Serbia

**Keywords:** autonomic nervous system dysfunction, chronic fatigue syndrome, post-COVID-19 syndrome, neurocardiology

## Abstract

**Background/Objectives:** Chronic fatigue syndrome and post-COVID-19 syndrome are associated with dysfunction of the autonomic nervous system, which may manifest as syncope and orthostatic intolerance. This study aimed to compare autonomic nervous system function in patients with chronic fatigue syndrome of unknown etiology and those with chronic fatigue syndrome secondary to post-COVID-19 syndrome using multiple diagnostic modalities, and to assess the prevalence and characteristics of syncope in these populations. **Methods:** This cross-sectional study included 440 patients examined at the Neurocardiological Laboratory of the Institute for Cardiovascular Diseases “Dedinje”. Patients were divided into three groups: chronic fatigue syndrome of unknown etiology (Group 1, n = 210), chronic fatigue syndrome secondary to post-COVID-19 syndrome (Group 2, n = 137), and healthy controls (Group 3, n = 91). Diagnostic modalities included cardiovascular reflex tests, the head-up tilt test, beat-to-beat analysis, 24 h Holter electrocardiogram monitoring, and 24 h ambulatory blood pressure monitoring. Statistical analyses were performed using analysis of variance, Tukey’s honestly significant difference test, and the Mann–Whitney U test. **Results:** Both chronic fatigue syndrome groups demonstrated significant autonomic nervous system dysfunction compared to healthy controls (*p* < 0.05), including reduced baroreceptor sensitivity and impaired heart rate variability parameters. Syncope prevalence was high in both chronic fatigue syndrome groups, with extreme blood pressure variability observed in 45–47% of patients during the head-up tilt test. Patients with post-COVID-19 chronic fatigue syndrome exhibited greater blood pressure increases during the head-up tilt test than those with chronic fatigue syndrome of unknown etiology (*p* < 0.05). **Conclusions:** Patients with chronic fatigue syndrome, irrespective of etiology, exhibit significant autonomic nervous system dysfunction and a high prevalence of syncope. Post-COVID-19 chronic fatigue syndrome demonstrates distinct hemodynamic patterns, suggesting unique pathophysiological mechanisms that warrant further investigation.

## 1. Introduction

Following the SARS-CoV-2 pandemic (COVID-19), there has been an increasing prevalence of post-COVID-19 syndrome, characterized by a wide range of clinical symptoms and a lack of a universally accepted definition. One proposed definition describes post-COVID-19 syndrome as a condition marked by the persistence of symptoms lasting more than four weeks after the acute phase of infection [1,2]. In parallel, chronic fatigue syndrome (CFS), also known as myalgic encephalomyelitis (ME), is recognized as a complex, chronic, multisystem disease with diverse constitutional and neurocognitive manifestations. The prevalence of CFS is estimated to be 0.17–0.89% in the general population, with a higher incidence among women [3]. The hallmark features of CFS include chronic fatigue persisting for more than six months, post-exertional malaise (PEM), cognitive dysfunction, sleep disturbances, orthostatic intolerance, and other systemic symptoms.

It is estimated that approximately 10% of individuals who experienced acute COVID-19 infection will develop CFS, with a hazard ratio of 4.32. Interestingly, the development of CFS appears to occur independently of the severity of the acute infection [4,5,6]. Notably, CFS can also emerge following other acute infections, such as Epstein–Barr virus (EBV) and *Coxiella burnetii* infections [7]. Although the pathophysiological mechanisms underlying both post-COVID-19 syndrome and CFS remain unclear, dysfunction of the autonomic nervous system (ANS) is believed to play a central role in the disease process. ANS dysfunction in patients with CFS has been strongly linked to disease severity [8,9].

Autonomic dysfunction is also a well-documented feature in syncope, including both neurally mediated and orthostatic syncope. Cases of syncope have been observed during acute COVID-19 infection as well as in post-COVID-19 syndrome [10,11]. Moreover, orthostatic intolerance and syncope are frequently reported in patients with CFS, with a prevalence ranging from 45% to 90% [12]. Notably, Kenny et al. reported that 21% of individuals with vasovagal syncope (VVS) met the diagnostic criteria for CFS, suggesting a potential overlap in the underlying pathophysiology of these conditions [13]. Similarly to CFS, where infection is often implicated as a primary trigger (e.g., COVID-19, EBV, *Coxiella burnetii*), the literature suggests that infections may also contribute to ANS dysfunction. Furthermore, the ANS has been proposed as a potential site of viral latency, raising questions about its role in the pathogenesis of both CFS and related syndromes [14,15,16,17].

The aim of this study is to investigate the function of the autonomic nervous system through functional diagnostic evaluations in patients with recurrent syncope and CFS. Specifically, we aim to explore CFS cases with an unidentified infectious etiology and those that developed as a consequence of post-COVID-19 syndrome.

## 2. Materials and Methods

This cross-sectional study included 440 patients aged 18 and older (19–70) who were examined in the Neurocardiological Laboratory of the Cardiology Clinic at the Institute for Cardiovascular Diseases, “Dedinje”. The patients were divided into three groups based on specific inclusion criteria. Group 1 consisted of 210 patients with recurrent syncope and a diagnosis of chronic fatigue syndrome (CFS) with insidious onset (mean age 44.85 ± 12.67, 43 males, and 167 females). Group 2 included 137 patients with recurrent syncope (two or more episodes in the past two months) and a diagnosis of CFS that developed after COVID-19 infection, corresponding to post-COVID-19 syndrome (mean age 45.02 ± 13.36, 33 males, and 104 females). Group 3 served as the healthy control group, consisting of 91 individuals with no history of syncope, CFS, or post-COVID-19 syndrome (mean age 36.6 ± 11.43, 37 males, and 54 females) (Table 1).

The diagnosis of chronic fatigue syndrome (CFS) was established using the 2021 criteria from the National Institute for Health and Care Excellence (NICE) guidelines [18]. According to these criteria, the diagnosis requires: (1) debilitating fatigue that worsens with activity, is not caused by excessive physical, cognitive, emotional, or social exertion, and is not significantly relieved by rest; (2) post-exertional malaise following activity; (3) unrefreshing sleep or sleep disturbances (or both), which may include flu-like exhaustion upon waking, stiffness, disrupted or shallow sleep, altered sleep patterns, or hypersomnia; and (4) cognitive difficulties (“brain fog”). The exclusion criteria for CFS include ruling out other potential causes, such as endocrine/metabolic disorders, cardiovascular and neurological conditions, primary anxiety and depression, and sleep disorders like sleep apnea.

The diagnosis of COVID-19 was confirmed based on WHO guidelines using a real-time reverse transcription–polymerase chain reaction (RT-PCR) assay [19]. Post-COVID-19 syndrome was defined as the continuation or development of new symptoms 3 months after the initial SARS-CoV-2 infection, with these symptoms lasting for at least 2 months with no other explanation, according to the WHO (World Health Organization) [19]. The diagnosis of CFS was established using the 2021 criteria from the National Institute for Health and Care Excellence (NICE) [18]. Exclusion criteria included patients with a cardiogenic etiology of syncope, such as conduction disorders, arrhythmia, congestive, ischemic, or valvular heart disease, or cardiomyopathy. Patients with transient loss of consciousness due to neurological causes, such as epilepsy confirmed by a neurology specialist, were also excluded. Additional exclusions encompassed orthostatic hypotension caused by volume depletion or medication, as well as primary autonomic neuropathies, including multiple system atrophy, pure autonomic failure, and Parkinson’s disease. Patients with abnormalities identified during 24 h Holter ECG monitoring, including conduction disorders necessitating pacemaker implantation, supraventricular tachycardia requiring ablation, or ventricular tachycardia, were also excluded.

This study was supported by grant 451-03-68/2020-14/200156 from the Ministry of Education, Science, and Technological Development of the Republic of Serbia and the grant COVANSA from the Science Fund of the Republic of Serbia. The study was approved by the Ethics Committee of the Institute for Cardiovascular Diseases “Dedinje” (No. 7548 approved on 13 December 2023). This study was performed in line with the principles of the Declaration of Helsinki.

### 2.1. Study Protocol

All participants underwent a comprehensive set of diagnostic evaluations to assess autonomic nervous system (ANS) function (Supplement S1). Cardiovascular reflex tests (CART) were performed in accordance with Ewing’s protocol [20]. These tests included assessments of sympathetic function, such as the hand grip test and blood response to standing, as well as evaluations of parasympathetic function using the Valsalva maneuver, heart response to breathing, and heart response to standing. In cases where heart response to standing and blood response to standing were assessed, they were performed during the head-up tilt test (HUTT). Each test was categorized as normal, borderline, or abnormal, with corresponding scores of 0, 1, and 2, respectively [21]. These scores were summed to calculate a total CART score ranging from 0 to 10.

The HUTT was conducted according to the Westminster protocol [22]. Before the passive phase of the test, patients rested in the supine position for 10 min. During the passive phase, patients were tilted to a 70° angle for a maximum of 30 min. A test was considered positive if syncope or severe presyncope occurred. Blood pressure and 12-lead ECG were continuously monitored throughout the procedure. The HUTT was not performed on individuals in the control group. To further assess autonomic function, the Task Force Monitor system was used to measure beat-to-beat heart rate and blood pressure variability [23]. The system performed spectral analysis of heart rate and blood pressure using an autoregressive methodology, providing data for parameters such as power spectral density, frequency bands (VLF, LF, HF), baroreceptor reflex sensitivity (BRS), and baroreceptor effectiveness index (BEI) [24,25]. Parameters were measured during the supine position phase and the passive phase of the HUTT. The Δ values, representing differences between the passive phase and supine position for each parameter, were also calculated.

Twenty-four-hour Holter ECG monitoring was performed using a 12-lead electrocardiogram device, and the recordings were analyzed by experienced analysts. After manual correction of artifacts and beat classifications, analyses of heart rate variability, QT and QTc intervals, deceleration capacity, acceleration capacity, and heart rate turbulence were conducted. Rhythm analysis included assessments of premature ventricular and atrial contractions. Parameters related to deceleration and acceleration capacities and heart rate turbulence could not be calculated for the control group due to the unavailability of the required software at the time of testing.

Twenty-four-hour ambulatory blood pressure monitoring was conducted using an oscillometric method with data collected every 15 min throughout the day. Variables analyzed included total, daytime, and nighttime systolic and diastolic blood pressure, mean arterial pressure, and pulse pressure.

### 2.2. Statistical Analysis

All results were presented as means with 95% confidence intervals or as counts and percentages, depending on the data type. Δ values were presented as mean differences. Data normality was assessed using the Smirnov test and normal Q-Q plots. Non-normally distributed data were transformed using logarithmic or square-root methods based on skewness and kurtosis. Transformed data were used for statistical analyses, with back-transformation performed for presentation. Between-group comparisons were made using ANOVA with Tukey’s HSD post hoc tests for parametric data, while nonparametric data were analyzed using chi-square and Mann–Whitney U tests. Statistical analyses were performed using SPSS version 26.0, with the level of significance set at *p* < 0.05.

## 3. Results

### 3.1. Head-Up Tilt Test (HUTT)

A positive HUTT was observed in 147 patients (71%) in Group 1 and in 97 patients (70.8%) in Group 2, with no statistically significant difference found between the groups (*p* > 0.05). Extreme blood pressure variations (where the absolute value of the difference between the maximum and minimum values of systolic pressure during variation is more than 20 mmHg) during the HUTT occurred in 94 patients (45.4%) in Group 1 and in 64 patients (46.7%) in Group 2, which was also not statistically significant (*p* > 0.05). Group 2 demonstrated a higher percentage of individuals with postural orthostatic tachycardia syndrome (POTS) (n = 7, 5.1%) compared to Group 1 (n = 8, 3.9%), but this difference did not reach statistical significance (*p* > 0.05).

### 3.2. Cardiovascular Reflex Tests (CART)

For the hand grip test, blood response to standing, heart response to breathing, and heart response to standing, Groups 1 and 2 exhibited significantly higher percentages of abnormal results compared to the control group (*p* < 0.05). However, there was no significant difference in the percentage of patients with abnormal Valsalva maneuver results between the control group (n = 17, 24.6%) and Group 1 (n = 68, 32.5%; *p* > 0.05). Group 1 demonstrated a significantly higher percentage of abnormal heart response to breathing results (n = 168, 80.4%) compared to Group 2 (n = 95, 70.4%; *p* < 0.05). Both sympathetic and parasympathetic dysfunctions were more prevalent in Groups 1 and 2 compared to the control group (*p* < 0.05) (Table 2).

### 3.3. Beat-to-Beat Analysis Using the Task Force Monitor

In the supine position, heart rate (HR) was significantly higher in Groups 1 and 2 compared to the control group (*p* < 0.05). Conversely, the diastolic blood pressure (DBP) in the supine position was significantly lower in Groups 1 and 2 compared to the control group (*p* < 0.05), while systolic blood pressure (SBP) was significantly lower in Group 1 compared to the control group (*p* < 0.05) (Table 3).

Spectral parameters of heart rate variability (HRV), including very low frequency (VLF), low frequency (LF), high frequency (HF), power spectral density (PSD), and baroreceptor effectiveness index (BEI), were significantly lower in Groups 1 and 2 compared to the control group (*p* < 0.05). Baroreceptor reflex sensitivity (BRS) was significantly lower in Group 1 compared to the control group (*p* < 0.05), while Group 2 exhibited lower BRS values without statistical significance (*p* > 0.05) (Table 3 and Table 4).

**Figure 1:**
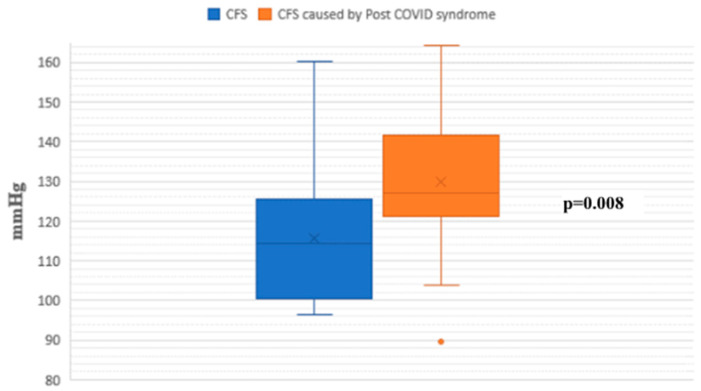
Systolic blood pressure during the passive phase of HUTT between groups.

**Figure 2:**
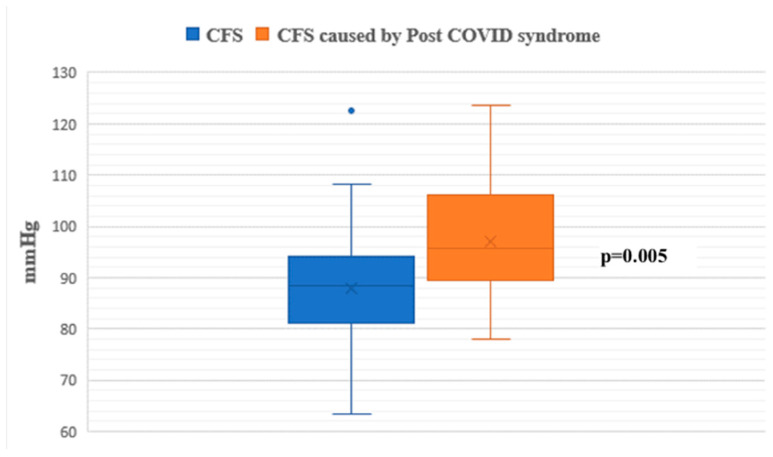
Diastolic blood pressure during the passive phase of HUTT between groups.

**Figure 3:**
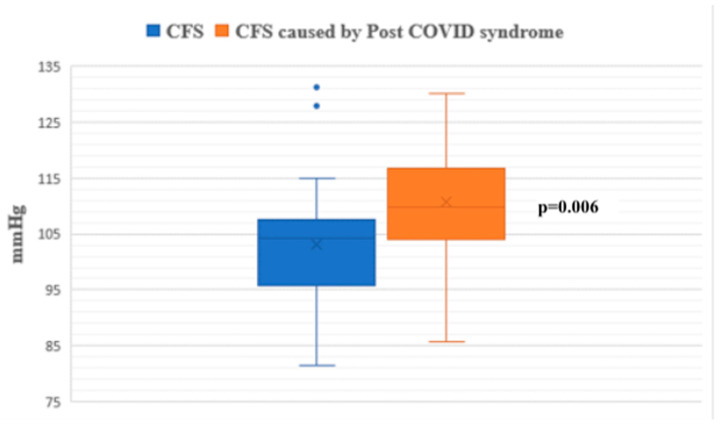
Mean arterial pressure during the passive phase of HUTT between groups.

During the passive phase of the HUTT, Group 1 demonstrated lower average SBP, DBP, and mean arterial pressure (MAP) values compared to Group 2 (*p* < 0.05) (Figure 1, Figure 2 and Figure 3).

**Figure 4:**
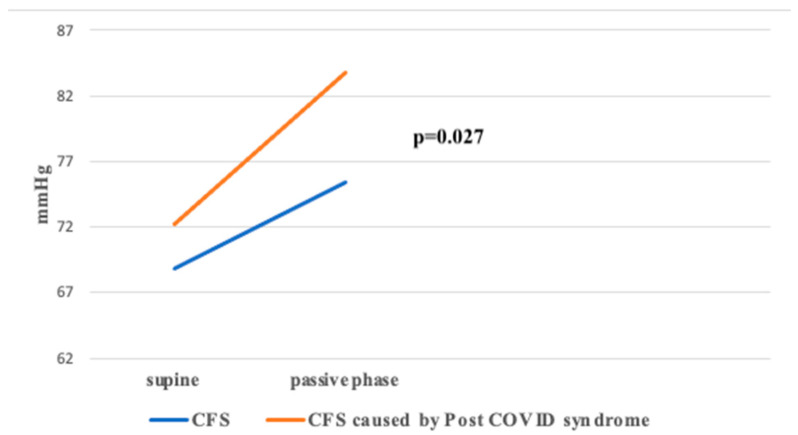
ΔDBP between groups during HUTT.

The ΔDBP value, representing the difference between passive and supine phases, was significantly higher in Group 2 compared to Group 1 (*p* < 0.05) (Figure 4).

No significant differences were observed in Δ values for spectral HRV parameters, BRS, or BEI (*p* > 0.05).

### 3.4. 24-Hour Holter ECG Monitoring

Parameters such as the standard deviation of the average NN intervals (SDANN), the percentage of NN intervals differing by more than 50 ms (pNN50), and high- and low-frequency HRV components (HF and LF, expressed in ms^2^) were significantly lower in Groups 1 and 2 compared to the control group (*p* < 0.05). Group 1 also exhibited significantly lower values of total power (TP), very low frequency (VLF), and the mean of the standard deviations of all NN intervals (SDNN) compared to the control group (*p* < 0.05) (Table 5).

Conversely, the LF/HF ratio was significantly higher in Groups 1 and 2 compared to the control group (*p* < 0.05). The deceleration capacity (DC) was significantly lower in Group 1 compared to Group 2 (*p* < 0.05). Interestingly, the control group had a significantly higher number of premature atrial contractions (PACs) compared to both patient groups (*p* < 0.05), while Groups 1 and 2 demonstrated significantly higher numbers of premature ventricular contractions (PVCs) compared to the control group (*p* < 0.05) (Table 6).

### 3.5. 24-Hour Ambulatory Blood Pressure Monitoring

**Figure 5:**
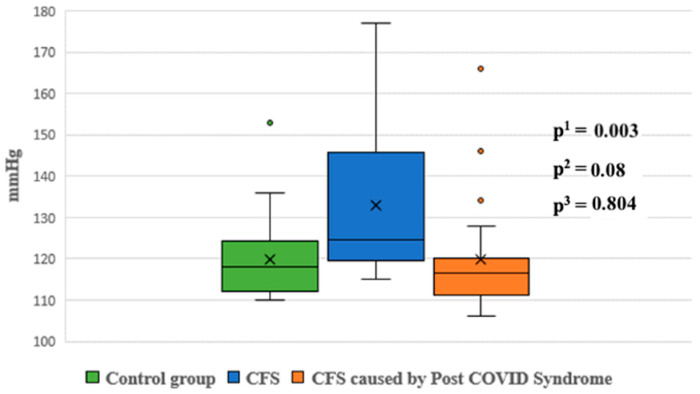
Systolic blood pressure at night between groups. p^1^—*p* value for comparison between control group and Group 1; p^2^—*p* value for comparison between control group and Group 2; p^3^—*p* value for comparison between Group 1 and Group 2.

**Figure 6:**
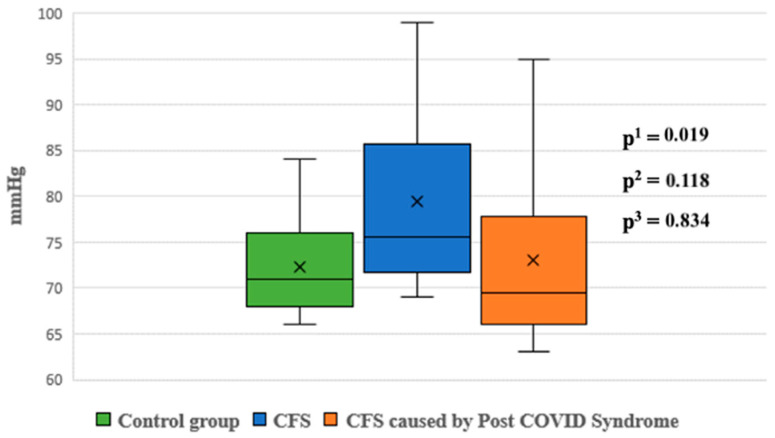
Diastolic blood pressure at night between groups. p^1^—*p* value for comparison between control group and Group 1; p^2^—*p* value for comparison between control group and Group 2; p^3^—*p* value for comparison between Group 1 and Group 2.

Night-time systolic and diastolic blood pressure levels were significantly higher in Group 1 compared to the control group (*p* < 0.05). No statistically significant differences were observed in other blood pressure parameters between the groups (Figure 5 and Figure 6).

## 4. Discussion

Although there was no statistically significant difference between the groups in the head-up tilt test (HUTT) outcomes, it is notable that both groups exhibited high percentages of positive HUTT results, with 71% in Group 1 and 70.8% in Group 2. These findings are consistent with the results of Bowe-Holaigah et al., who reported a 70% prevalence of abnormal HUTT in patients with chronic fatigue syndrome (CFS) during the initial phase of the test without isoproterenol use [12]. Additionally, 45–47% of subjects, depending on the group, exhibited extreme variations in blood pressure during HUTT, defined as fluctuations exceeding 20 mmHg. This phenomenon may contribute to symptoms of orthostatic intolerance, which is characteristic of CFS [12]. Moreover, Hausenloy et al. identified an 87% positive predictive value for vasovagal syncope during HUTT in cases with extreme pressure variations, further reinforcing the relevance of this finding [26]. In Group 2, 5.1% of patients were diagnosed with postural orthostatic tachycardia syndrome (POTS), compared to 3.9% in Group 1, aligning with observations by van Campen et al., who noted higher POTS prevalence in patients with CFS secondary to COVID-19 infection [27]. Furthermore, van Campen et al. reported an abnormal reduction in cerebral blood flow in 90% of CFS patients during HUTT, regardless of hemodynamic changes such as POTS or orthostatic hypotension [28].

Cardiovascular reflex tests (CART) revealed both sympathetic and parasympathetic dysfunction in Groups 1 and 2, with statistically significant differences compared to the control group (*p* < 0.05). While Group 1 demonstrated slightly higher percentages of sympathetic dysfunction, parasympathetic dysfunction, and complete autonomic dysfunction compared to Group 2, these differences were not statistically significant (*p* > 0.05). Orthostatic hypotension (OH), defined as a pressure drop exceeding 30 mmHg and a key marker of sympathetic dysfunction, was more prevalent in Group 1. This finding suggests a greater occurrence of syncope due to orthostatic hypotension in CFS unrelated to post-COVID-19 syndrome. The higher prevalence of abnormal OH tests in Group 1 also resulted in a higher autonomic neuropathy score, though the difference was not statistically significant. Notably, patients in Group 2 exhibited the highest percentage of abnormal responses to the Valsalva maneuver, which was statistically significant compared to the control group, but which was not observed in Group 1. Similar findings were reported by Milovanović et al., who documented combined autonomic nervous system (ANS) damage in acute COVID-19 infections compared to healthy controls [29].

**Figure 7:**
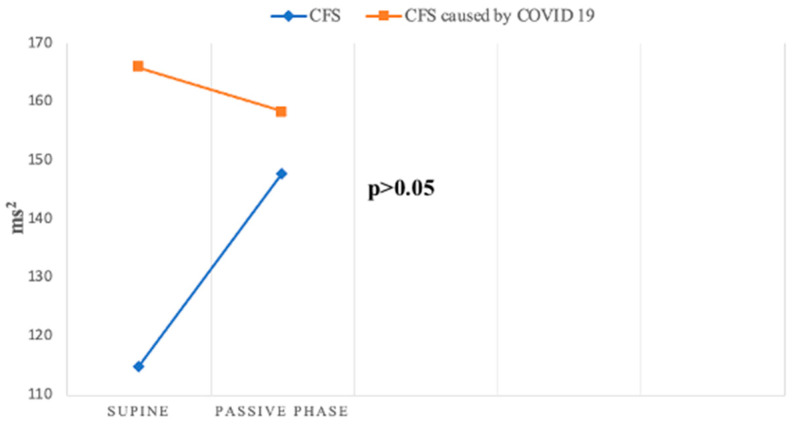
Change in HF—RRI during HUTT between groups.

Beat-to-beat analysis showed that spectral parameters of heart rate variability (HRV) in the supine position were significantly lower in both groups compared to the control group, indicating reduced activity in both sympathetic and parasympathetic components of the ANS. These findings align with the work of Ryabkov et al., who also observed reduced HRV parameters in patients with CFS and post-COVID-19 syndrome [30]. During the passive phase of the HUTT, there was a different pattern of HF—RRI changes during the passive phase between two groups (Figure 7).

However, HF, a key marker of parasympathetic function, exhibited divergent patterns: it decreased in Group 2 but increased in Group 1. This discrepancy suggests distinct parasympathetic response patterns between the two groups, as confirmed by Da Silva et al., who also reported decreased HF values during HUTT in patients with post-COVID-19 syndrome [31].

**Figure 8:**
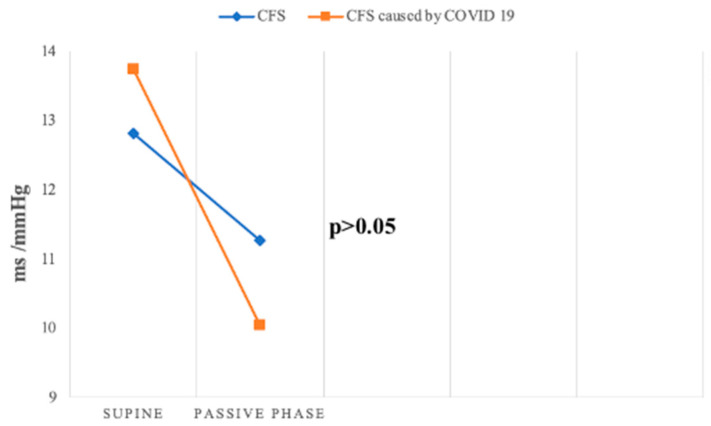
Change in BRS slope mean during passive HUTT between groups.

Baroreceptor reflex sensitivity (BRS) and the baroreceptor effectiveness index (BEI) were lower in both groups compared to the control group, indicating impaired baroreceptor function, a crucial mechanism for short-term blood pressure regulation. Both parameters decreased further during the passive phase of the HUTT in Groups 1 and 2. Peckerman et al. similarly observed a reduction in BRS during HUTT in patients with severe CFS [32]. Although BRS and BEI values were higher in the supine position in Group 2 compared to Group 1, they decreased more significantly during the passive phase in Group 2, highlighting greater baroreceptor impairment in this group (Figure 8).

Milovanović et al. and Srivastava et al. similarly reported decreased BRS in patients with acute and post-acute COVID-19 infection, respectively, emphasizing baroreceptor involvement in autonomic dysfunction associated with COVID-19 [29,33].

During 24 h Holter ECG monitoring, both groups exhibited significantly reduced HRV parameters, including total power, VLF, LF, pNN50, and HF compared to the control group, indicating dysfunction in both the sympathetic and parasympathetic branches of the ANS. Interestingly, Group 1 showed the lowest total power and VLF values, differing significantly from the control group, suggesting more pronounced autonomic dysfunction. Despite this, the LF/HF ratio was higher in both groups compared to the control group, indicating sympathetic dominance. DC values, indicative of vagal influence on the heart, were significantly lower in Group 1 compared to Group 2, suggesting more severe parasympathetic dysfunction in Group 1 [34]. Walitt et al. similarly reported reduced HRV parameters and diminished nocturnal HR drops in patients with CFS, consistent with the findings of this study [35].

Heart rate turbulence (HRT) parameters, indicative of baroreflex-mediated heart rate adjustments following premature contractions, did not differ significantly between the two groups (Table 3). However, abnormal turbulence onset (TO) values were observed in Group 2, with average TO values exceeding the normal range, suggesting vagal inhibition following PVCs. Similar findings were reported for HRT related to PACs, though physiological mechanisms may differ [36].

Ambulatory blood pressure monitoring revealed that night-time systolic and diastolic pressures were significantly higher in Group 1 compared to the control group (*p* < 0.05). Although Group 1 also showed higher night-time pressures compared to Group 2, these differences were not statistically significant. Newton et al. similarly reported lower daytime systolic pressures in patients with CFS compared to controls, further supporting these findings [37].

Overall, the results demonstrate significant autonomic dysfunction in patients with CFS, both related and unrelated to post-COVID-19 syndrome. The findings align with previous studies suggesting that infections, including SARS-CoV-2, may precipitate ANS dysfunction and contribute to the pathophysiology of CFS and post-COVID-19 syndrome [4,5,6,38]. The dysregulation of the immune system, increased oxidative stress, intestinal flora disruption, and viral reactivation are proposed mechanisms linking infections to CFS [38,39,40,41,42]. Aside from the connection between CFS and infection, numerous microorganisms were associated with autonomic nervous system impairment, such as HSV, Coxsackiae, CMV, EBV, and Borrelia spp. [43,44,45,46,47,48,49,50,51,52,53,54,55,56,57,58,59]. Moreover, the interplay between the immune and autonomic systems, including the role of β-adrenergic and muscarinic receptor antibodies, may further elucidate the pathogenesis of these conditions [60,61,62,63,64,65,66,67]. This study underscores the critical role of the ANS in the development of syncope, CFS, and post-COVID-19 syndrome, and highlights the potential shared mechanisms underlying these disorders.

### Study Limitations

One limitation of this study is that parameters such as deceleration capacity (DC), acceleration capacity (AC), heart rate turbulence (HRT), and spectral domains of HRV, along with baroreceptor reflex sensitivity (BRS) and baroreceptor effectiveness index (BEI) were not measured in the healthy control group during the passive phase of the HUTT. This omission precluded the direct comparison of these specific parameters between the control group and the study groups, which could have provided additional insights into the autonomic differences between these populations. Furthermore, it is important to note that many patients with ME/CFS do not have recurrent syncope, and therefore were not included in this study, Thus, future studies should also include this population and the assessment of lung function as well.

## 5. Conclusions

A significant proportion of patients with chronic fatigue syndrome (CFS), including those with post-COVID-19 syndrome, experience episodes of syncope during the course of the disease. Syncope in these patients, similar to CFS itself, is characterized by autonomic nervous system dysfunction. Patients with post-COVID-19 CFS exhibited a greater increase in blood pressure during the head-up tilt test (HUTT) compared to those with CFS of unknown infectious origin. Despite this difference, both groups demonstrated comparable degrees and types of autonomic dysfunction across multiple diagnostic modalities, including cardiovascular reflex tests (CART), beat-to-beat analysis, 24 h Holter ECG monitoring, and 24 h ambulatory blood pressure monitoring. Both groups exhibited significant autonomic impairment when compared to healthy controls, emphasizing the role of autonomic dysfunction in the pathophysiology of both CFS and syncope.

## Figures and Tables

**Table 1:** Study population demographic characteristics.

	CFS N = 210	CFS After COVID-19 N = 137	Control Groups N = 91
Years (mean ± SD)	44.85 ± 12.67	45.02 ± 13.36	36.6 ± 11.43
Male (n, %)	43 (20.48%)	33 (24.09%)	37 (40.65%)
Female (n, %)	167 (79.52%)	104 (75.91%)	54 (59.35%)
Sleep disturbances (n, %)	210 (100%)	137 (100%)	0 (0%)
PEM (n, %)	210 (100%)	137 (100%)	0 (0%)
Fibromylagia (n, %)	154 (73.3%)	86 (62.8%)	0 (0%)
Joint pain (n, %)	154 (73.3%)	89 (65%)	0 (0%)
Vertigo (n, %)	94 (44.8%)	58 (42.3%)	0 (0%)
Disturbed regulation of temperature (n, %)	98 (46.7%)	18 (13.1%)	0 (0%)
Tingling (n, %)	113 (53.8%)	50 (36.5%)	0 (0%)
Burning sensation (n, %)	128 (61%)	36 (26.3%)	0 (0%)

**Table 2:** Result of cardiovascular reflex tests by Ewing.

Groups	Control Group (n = 69)	CFS (n = 210)	CFS Caused by Post-COVID-19 Syndrome (n = 135)	Sig.
HGT * n (%)	51 (73.9%)	206 (96.1%)	131 (97%)	p^1^ < 0.001 p^2^ < 0.001 p^3^ > 0.05
OH n (%)	0 (0%)	73 (34.8%)	34 (25.2%)	p^1^ < 0.001 p^2^ < 0.001 p^3^ > 0.05
DS n (%)	51 (73.9%)	207 (98.6%)	131 (97.0%)	p^1^ < 0.001 p^2^ < 0.001 p^3^ > 0.05
VM n (%)	17 (24.6%)	68 (32.5%)	51 (37.8%)	p^1^ > 0.05 p^2^ < 0.05 p^3^ > 0.05
HRB n (%)	11 (15.9%)	168 (80.4%)	95 (70.4%)	p^1^ < 0.001 p^2^ < 0.001 p^3^ < 0.05
HRS n (%)	36 (52.2%)	195 (93.3%)	118 (84.4%)	p^1^ < 0.001 p^2^ < 0.001 p^3^ > 0.05
DP (definite) n (%)	11 (15.9%)	167 (79.9%)	104 (77%)	p^1^ < 0.001 p^2^ < 0.001 p^3^ > 0.05
CAN n (%)	38 (55.1%)	207 (97.6%)	129 (95.6%)	p^1^ < 0.001 p^2^ < 0.001 p^3^ > 0.05
Score	4.33 (3.98–4.69)	7.25 (7.04–7.47)	6.96 (6.71–7.2)	p^1^ < 0.001 p^2^ < 0.001 p^3^ > 0.05

* For HGT, OH, VM, HRB, and HRS, only abnormal results of tests were shown in Table 2. CART—cardiovascular reflex tests; HGT—hand grip test; OH—orthostatic hypotension test (blood pressure response to standing); DS—sympathetic disfunction; VM—Heart response to Valsalva maneuver; HRB—heart response to breathing; HRS—heart response to standing; DP—parasympathetic dysfunction; CAN—complete autonomic neuropathy; p^1^—*p* value for comparison between control group and Group 1; p^2^—*p* value for comparison between control group and Group 2; p^3^—*p* value for comparison between Group 1 and Group 2.

**Table 3:** Beat-to-beat analysis of blood pressure, BRS, and BEI in the supine position.

Parameters	Control Group (n = 91)	CFS (n = 68)	CFS Caused by Post-COVID-19 (n = 60)	Sig.
HR (bpm)	72.32 (70.14–74.5)	77.46 (74.2–81.09)	80.91 (76.82–85)	p^1^ < 0.05 p^2^ < 0.001 p^3^ > 0.05
SBP (mmHg)	115.9 (113.12–118.66)	107.14 (103.39–110.88)	111.84 (108.01–115.67)	p^1^ < 0.01 p^2^ > 0.05 p^3^ > 0.05
DBP (mmHg)	76.87 (74.44–79)	68.88 (65.64–72.12)	72.28 (69.3–75.25)	p^1^ < 0.001 p^2^ < 0.05 p^3^ > 0.05
MAP (mmHg)	88.86 (86.71–90.1)	84.48 (81.18–87.77)	88.15 (85.03–91.26)	p^1^ > 0.05 p^2^ > 0.05 p^3^ > 0.05
BRS (ms/mmHg)	17.9 (15.84–19.95)	12.82 (10.17–15.46)	13.75 (11.09–16.41)	p^1^ < 0.01 p^2^ > 0.05 p^3^ > 0.05
BEI (%)	121.12 (111.87–130.38)	61.84 (54.10–69.57)	64.41 (58.45–70.36)	p^1^ < 0.001 p^2^ < 0.001 p^3^ > 0.05

HR—heart rate; bpm—beat per minute; SBP—systolic blood pressure; mmHg—millimeters of mercury; DBP—diastolic blood pressure; MAP—mean arterial pressure; BRS—baroreflex sensitivity; ms/mmHg—milliseconds per millimeters of mercury; BEI—baroreflex sensitivity index; p^1^—*p* value for comparison between control group and Group 1; p^2^—*p* value for comparison between control group and Group 2; p^3^—*p* value for comparison between Group 1 and Group 2.

**Table 4:** Beat-to-beat analysis of spectral parameters of HRV during the supine position.

Parameters	Control Group (n = 91)	CFS (n = 68)	CFS Caused by Post-COVID-19 (n = 60)	Sig.
LFnu-RRI (%)	59.65 (56.25–63.05)	62.95 (58.55–67.35)	60.13 (55.18–65.08)	p^1^ > 0.05 p^2^ > 0.05 p^3^ > 0.05
HFnu-RRI (%)	40.02 (36.77–43.27)	37.65 (33.16–42.15)	39.87 (34.92–44.83)	p^1^ > 0.05 p^2^ > 0.05 p^3^ > 0.05
PSD-RRI (ms^2^)	1258.93 (977.24–1621.81)	630.96 (467.74–851.14)	645.65 (457.09–912.01)	p^1^ < 0.01 p^2^ < 0.01 p^3^ > 0.05
VLF-RRI (ms^2^)	257.04 (204.17–323.59)	154.88 (112.20–213.80)	134.90 (95.50–190.55)	p^1^ < 0.05 p^2^ < 0.01 p^3^ > 0.05
LF-RRI (ms^2^)	512.86 (407.38–645.65)	223.87 (169.82–295.12)	263.03 (190.55–363.08)	p^1^ < 0.001 p^2^ < 0.01 p^3^ > 0.05
HF-RRI (ms^2^)	316.23 (239.88–416.87)	114.82 (79.43–165.96)	165.96 (107.15–257.04)	p^1^ < 0.001 p^2^ < 0.05 p^3^ > 0.05
LFnu/HFnu-RRI	1.91 (1.58–2.29)	2.14 (1.66–2.75)	1.74 (1.38–2.19)	p^1^ > 0.05 p^2^ > 0.05 p^3^ > 0.05
LF/HF-RRI	1.55 (1.32–1.82)	1.41 (1.12–1.78)	1.29 (1.02–1.62)	p^1^ > 0.05 p^2^ > 0.05 p^3^ > 0.05

LFnu-RRI—normalized low frequency of HRV; Hfnu-RRI—normalized high frequency of HRV; PSD-RRI—power spectral density of HRV; VLF-RRI–very-low-frequency component of HRV; LF-RRI—low-frequency component of HRV; HF-RRI—high-frequency component of HRV; LFnu/Hfnu-RRI—normalized low frequency/normalized high frequency ratio of HRV; LF/HF-RRI—low frequency/high frequency ratio of HRV; p^1^—*p* value for comparison between control group and Group 1; p^2^—*p* value for comparison between control group and Group 2; p^3^—*p* value for comparison between Group 1 and Group 2.

**Table 5:** Heart rate, RR interval and time domain parameters of HRV during 24 h Holter ECG monitoring.

Groups	Control Group (n = 86)	CFS (n = 64)	CFS Caused by Post-COVID-19 (n = 37)	Sig.
Heart rate (bpm)	75.19 (73.37–77.00)	73.83 (71.61–76.04)	74.84 (71.95–77.26)	p^1^ > 0.05 p^2^ > 0.05 p^3^ > 0.05
RR interval (ms)	816.52 (794.33–829.09)	811.32 (787.65–835)	801.2 (770.28–832.13)	p^1^ > 0.05 p^2^ > 0.05 p^3^ > 0.05
SDNN (ms)	159.15 (151.44—166.85)	145.94 (137.54–154.34)	144.76 (132.01–157.51)	p^1^ > 0.05 p^2^ > 0.05 p^3^ > 0.05
SDANN (ms)	145.50 (137.41–153.59)	130.33 (122.47–138.2)	128.76 (115.85–141.67)	p^1^ < 0.05 p^2^ < 0.05 p^3^ > 0.05
SDNNIN (ms)	64.57 (60.26–69.18)	54.95 (52.48–57.54)	61.66 (52.48–72.44)	p^1^ < 0.05 p^2^ > 0.05 p^3^ > 0.05
rMSSD (ms)	38.65 (35.31–42.00)	40.35 (35.15–45.54)	37.3 (31.54–43.05)	p^1^ > 0.05 p^2^ > 0.05 p^3^ > 0.05
pNN50 (%)	11,75 (10.00–13.80)	7.94 (6.46–9.77)	6.61 (4.79–9.12)	p^1^ < 0.01 p^2^ < 0.05 p^3^ > 0.05

SDNN—standard deviation of normal RR intervals; ms—milliseconds; SDANN—standard deviation of the average NN intervals for each 5 min segment of a 24 h HRV recording; SDNIN—mean of the standard deviations of all NN intervals for each 5 min segment of a 24 h HRV recording; rMSSD—root mean square of successive differences; pNN50—the percentage of intervals > 50 ms different from preceding interval; p^1^—*p* value for comparison between control group and Group 1; p^2^—*p* value for comparison between control group and Group 2; p^3^—*p* value for comparison between Group 1 and Group 2.

**Table 6:** Spectral parameters of HRV, DC, AC, and HRT parameters, along with the number of PVC/PAC during 24 h Holter ECG monitoring.

Groups	Control Group (n = 86)	CFS (n = 64)	CFS Caused by Post-COVID-19 (n = 37)	Sig.
TP (ms^2^)	4430.33 (3965.7–4894.96)	3143.19 (2724.77–3561.61)	3571.45 (2891.16–4251.75)	p^1^ < 0.01 p^2^ < 0.05 p^3^ > 0.05
VLF (ms^2^)	3012.07 (2659.27–3364.87)	2160.95 (1874.38–2447.53)	2408.60 (1925.15–2892.06)	p^1^ < 0.01 p^2^ > 0.05 p^3^ > 0.05
LF (ms^2^)	1053.97 (956.28–1151.66)	707.31 (600.05–814.58)	823.23 (650.67–995.78)	p^1^ < 0.001 p^2^ < 0.05 p^3^ > 0.05
HF (ms^2^)	331.13 (281.84–389.05)	204.17 (169.82–245.47)	229.09 (186.21–281.84)	p^1^ < 0.001 p^2^ < 0.05 p^3^ > 0.05
LF/HF	1.96 (1.89–2.03)	3.37 (2.87–3.88)	3.44 (2.87–4.0)	p^1^ < 0.01 p^2^ < 0.1 p^3^ > 0.05
PVC (mean + 95% interval)	59.16 (15.45–102.86)	184.37 (105.85–262.9)	247.38 (0–498.67)	p^1^ < 0.001 p^2^ < 0.01 p^3^ > 0.05
PAC (mean + 95% interval)	97.71 (0–195.70)	68.69 (39.48–97.91)	69.27 (24.7–113.84)	p^1^ < 0.001 p^2^ < 0.001 p^3^ > 0.05
DC (ms)	/	6.5 (6.4–7.3)	7.54 (7.02–8.06)	p^3^ < 0.5
AC (ms)	/	7.51 (6.89–8.12)	8.25 (7.59–8.92)	p^3^ > 0.05
TO (PVC) (%)	/	−2 (3.83–−0.17)	3.38 (−5.24—1.52)	p^3^ > 0.05
TS (PVC) (ms/RR interval)	/	13.3 (7.85–18.74)	14.1 (9.86–18.34)	p^3^ > 0.05
TO (PAC) (%)	/	2.06 (0.34–3.77)	0.78 (−1.54–3.1)	p^3^ > 0.05
TS (PAC) (ms/RR interval)	/	6.77 (4.75–8.8)	7.57 (4.94–10.19)	p^3^ > 0.05

TP—total power of HRV; ms^2^—millisecond squared; VLF—very-low-frequency component of HRV; LF—low-frequency component of HRV; HF—high-frequency component of HRV; LF/HF—low frequency/high frequency ratio of HRV; PVC—premature ventricular contraction; PAC—premature atrial contraction; DC—deceleration capacity; AC—acceleration capacity; TO—turbulence onset; TS—turbulence slope; p^1^—*p* value for comparison between control group and Group 1; p^2^—*p* value for comparison between control group and Group 2; p^3^—*p* value for comparison between Group 1 and Group 2.

## Data Availability

Data are available upon reasonable request.

## Supplementary Materials

The following supporting information can be downloaded at: https://www.mdpi.com/article/10.3390/jcm14030811/s1, Supplement S1: Tests Conducted and Measurement Methodology.
