# Supplementary material for: Assessment of Autonomic Nervous System Function in Patients with Chronic Fatigue Syndrome and Post-COVID-19 Syndrome Presenting with Recurrent Syncope
Source: J Clin Med. 2025 Jan 26;14(3):811. doi: 10.3390/jcm14030811 (PMC11818862; doi:10.3390/jcm14030811)
Supplement: Supplementary file 1 [file jcm-14-00811-s001.zip › jcm-3372958-supplementary.pdf]

Supplement 1. Tests Conducted and Measurement Methodology

Test	Measurement Methodology
Cardiovascular Reflex Tests (CART)	Ewing's Protocol
Head Up Tilt Test (HUTT)	Westminster protocol
Blood Pressure (BP)	Task Force Monitor
Heart Rate Variability (HRV)	Task Force Monitor
Ambulatory Blood Pressure	Mobil–O–Graph blood pressure monitors
Long term HRV	DMS Software Scan
Deceleration and Acceleration Capacity	DMS Software Scan
Heart rate turbulence	DMS Software Scan
